# The Sustained Effects of Bioactive Collagen Peptides on Skin Health: A Randomized, Double‐Blind, Placebo‐Controlled Clinical Study

**DOI:** 10.1111/jocd.70565

**Published:** 2025-11-28

**Authors:** Yu Wang, Weixing Zhu, Wenyu Luo, Yanfeng Ma, Yue Zhou

**Affiliations:** ^1^ Department of Innovation R&D Shanghai Meifute Biotechnology Co., Ltd Shanghai China; ^2^ Department of Dermatology, 411 Hospital, School of Medicine Shanghai University Shanghai China; ^3^ Shanghai Fumei Medical Cosmetology Clinic Shanghai China

## Abstract

**Introduction:**

Collagen is a fundamental component of the skin's extracellular matrix, yet its low oral bioavailability raises questions about its efficacy in improving skin health. Bioactive collagen peptides (BCP), as hydrolyzed forms of collagen, offer enhanced absorption and functionality. However, evidence regarding their sustained effects and the impact of molecular weight distribution is still limited.

**Aim:**

To evaluate the long‐term effects of BCP supplementation on skin hydration, elasticity, and dermal structure, and to explore the relationship between molecular properties of BCP and skin outcomes.

**Methods:**

A randomized, double‐blind, placebo‐controlled trial was conducted involving 77 healthy female participants aged. Subjects received either 5000 mg/day of BCP or placebo for 12 weeks, followed by a 4‐week washout period. Skin parameters—including hydration, transepidermal water loss (TEWL), elasticity, and dermal/epidermal thickness and density—were measured at baseline, Week 8, Week 12, and Week 16 using non‐invasive techniques.

**Results:**

BCP significantly improved dermal density, hydration, and TEWL compared to placebo after 12 weeks (*p* < 0.05). These improvements were maintained during the washout period. Dermal thickness also increased in the BCP group, while no significant changes were noted in the epidermis.

**Conclusions:**

Twelve weeks of oral BCP supplementation, followed by a 4‐week washout, produced lasting improvements in skin hydration, firmness, and dermal structure. These effects are likely linked to the BCP's high hydroxyproline content and low molecular weight distribution, supporting its use in anti‐aging skincare strategies.

**Trial Registration:**

ClinicalTrials.gov identifier: NCT06847035

## 1 Introduction

Skin is the largest organ of the human body, and its structure and function play critical roles in barrier protection, immune regulation, and sensory perception of external stimulation [1, 2]. Skin health and appearance depend primarily on the quantity and quality of dermal collagen [3], which constitutes 70%–80% of dermal dry weight and whose integrity directly determines skin elasticity, firmness, and moisture‐holding capacity [4]. Among the collagen subtypes, type I collagen (COL I) was the predominant component, serving as the primary physical barrier between the environment and body fluids [5]. However, intrinsic aging and extrinsic factors such as ultraviolet (UV) irradiation and environmental pollution progressively damaged the dermal extracellular matrix [6], resulting in collagen degradation outpacing synthesis 2 and thereby compromising tissue structure and reparative capacity. Notably, from age 30 onward, dermal collagen content declined by 1%–2% per year, accompanied by disorganization and fragmentation of collagen fibers, ultimately leading to wrinkles, laxity, and reduced elasticity [7].

In recent years, collagen peptides have emerged as functional food supplements for improving skin health due to their favorable absorption and bioavailability [8]. BCP were defined as collagen‐derived peptides that have been shown in vitro to promote fibroblast proliferation [9, 10]. Multiple meta‐analyses have shown that collagen peptides can improve skin elasticity and hydration [11, 12, 13]. In vivo, BCP were absorbed as di‐and tripeptides, transported via the bloodstream to the dermis, where they stimulate synthesis of COL I and type III collagen (COL III) while inhibiting matrix metalloproteinase (MMP)‐mediated collagen degradation [14]. Mechanistic studies further demonstrated that BCP enhance skin function by regulating hyaluronic acid (HA) synthesis [15], exerting anti‐inflammatory [16] and antioxidant effects [17], and activating the transforming growth factor‐β1 (TGF‐β1)/Smad signaling pathway [18]. To date, most clinical trials have used raw BCP materials rather than commercially formulated products; however, commercial formulations ensure consistency of active ingredients and allow evaluation of synergistic effects with other nutrients [19].

Although continuous BCP intake has shown efficacy in several studies [2, 20, 21, 22], these investigations typically assess outcomes only during the intervention period, neglecting long‐term skin changes post‐cessation. Furthermore, few clinical studies specify the molecular‐weight distribution of the BCP raw material. The hydroxyproline content and molecular weight distribution were closely related to the source of collagen [23]. Therefore, in this study we selected a BCP raw material characterized by high hydroxyproline content and an optimized molecular‐weight profile, and conducted a clinical trial to evaluate its effects on skin hydration and elasticity. Distinct from prior systematic reviews and meta‐analyses [13], which rarely quantified both dermal thickness and density, we employed high‐resolution ultrasound imaging to include dermal thickness and density as core endpoints. This integrated approach provides new structural evidence for the mechanisms by which BCP enhance skin mechanical properties and offers theoretical and practical guidance for their scientific application and development as functional foods.

## 2 Materials and Methods

### 2.1 Study Design and Participants

This randomized, controlled, double‐blind, placebo‐controlled trial was conducted at Shanghai Fumei Medical Cosmetology Clinic. Over a 16‐week period, 83 healthy female participants (aged 35–55 years) were randomly assigned to study groups. Inclusion criteria required:

- Female gender;
- Good systemic health;
- No consumption of collagen peptide‐based functional foods within the past year;

Exclusion criteria comprised: Female participant

- Pregnancy, lactation, or plans for pregnancy;
- Perimenopausal status;
- Body mass index (BMI) < 18.5 or > 27.9;
- History of chronic systemic diseases (e.g., cardiovascular, cerebrovascular, hepatic, or renal disorders), diabetes, or dermatological conditions (e.g., psoriasis, eczema, atopic dermatitis, severe acne);
- Psychological conditions such as depression or sleep disorders;
- Current smoking, alcohol dependence, or use of hormone therapy, anti‐obesity medications, absorption inhibitors, antidepressants, or appetite suppressants;
- Recent medical aesthetic procedures (e.g., chemical peels, laser treatments) at the test site within 3 months.
- Topical application of hormonal or anti‐inflammatory agents at the test site within 2 months.
- Inability to avoid prolonged sun exposure;
- Participation in other clinical trials within 3 months.
- Any condition deemed clinically unsuitable by investigators.

The study protocol was approved by the Ethics Committee of [Institution Name Redacted] (Approval No.: number) and registered on ClinicalTrials.gov (NCT number). All participants provided written informed consent, and the study complied with the Declaration of Helsinki and ethical standards for participant privacy and data security. Participants were randomly assigned to the treated or placebo group using a computer‐generated sequence. Both participants and investigators were blinded to group allocation, with test products being indistinguishable in appearance, taste, and texture. The trial included four visits: baseline (Week 0) and follow‐ups at Weeks 8, 12, and 16. Participants received daily supplementation for 12 weeks, followed by a 4‐week discontinuation phase to assess sustained effects.

### 2.2 Research Products

This study employed a proprietary blend containing BCP (LISAVEI collagen peptide powder beverage; Shanghai, China), and the BCP was derived from bovine type I collagen via specific enzymatic hydrolysis. According to previous research, the clinical doses for skin‐health benefits range from 2.5 g to 5 g per day [24, 25] whereas doses of 10–20 g/day have been used to improve muscle function [26]. Therefore, participants were randomized to receive either 5000 mg of BCP or a matching placebo once daily, with the composition shown in Table 1. For consistency, the powder was dissolved each morning in 250 mL of water at room temperature. The intervention continued for 12 weeks, followed by a 4‐week washout period to evaluate the durability of any observed skin effects.

**TABLE 1:** Main ingredients of the product of research.

Main ingredients	Treated (mg)	Placebo (mg)
BCP	5000	0.00
Maltodextrin	0	5000
Vitamin C	12.6	12.6
Vitamin E	0.58 mg α‐TE	0.58 mg α‐TE
Total	5013.18	5013.18

### 2.3 Characterization of Bioactive Collagen Peptides

BCP sample was first dissolved in tetrahydrofuran and filtered to remove insoluble impurities. Molecular‐weight distribution was determined by gel‐permeation chromatography (GPC) using an EcoSEC HLC‐8320GPC system, equipped with a GPC column. The mobile phase consisted of acetonitrile: water: trifluoroacetic acid (40:60:0.05, v/v/v) at a flow rate of 0.5 mL/min, with the column maintained at 30°C. A 10 μL aliquot of each sample was injected, and peptide fractions were quantified against molecular‐weight standards to generate a distribution profile. Hydroxyproline content was determined using a commercial assay kit (Cat. No. A030‐3‐1, Nanjing Jiancheng Bioengineering Institute, Nanjing, China) based on the acid hydrolysis method, according to the manufacturer's instructions.

### 2.4 Clinical Measurements

All clinical efficacy measurements were performed in a controlled environment (relative humidity 40%–60%, room temperature 20°C–24°C). Subjects rested quietly and refrained from drinking for 1 h prior to assessment. Dermal thickness and density on the facial regions were evaluated by high‐frequency ultrasound (HFUS) (Ultrascan UC22; Courage + Khazaka electronic GmbH, Germany) at 20 MHz. Skin elasticity parameters—including overall elasticity (R2) and firmness (F4)—were measured with a Cutometer dual MPA 580 (Courage + Khazaka electronic GmbH, Germany). Facial hydration and transepidermal water loss (TEWL) were assessed using the Cutometer CM 825 (Courage + Khazaka electronic GmbH, Germany). Each measurement was repeated three times per site and averaged for analysis. Assessments were conducted at five facial sites: forehead, left cheek, right cheek, nose, and chin. Skin type was classified by dermatologists according to the *Chinese Facial Skin Classification and Skin Care Guide*.

### 2.5 Safety Assessment

Adverse reactions were assessed by dermatologists at the outpatient clinic. The safety evaluation included detailed interviews and physical examinations to identify any potential side effects or intolerances related to the intervention.

### 2.6 Statistical Analysis

Descriptive statistics were applied to summarize all collected data. Between‐group comparisons were conducted using two‐factor analysis of variance (ANOVA). All statistical analyses were performed using GraphPad Prism 8.0 software (GraphPad Software LLC, California, USA). A *p*‐value of < 0.05 was considered indicative of statistical significance.

## 3 Results

### 3.1 Trial Recruitment and Follow‐Up Process

**FIGURE 1:**
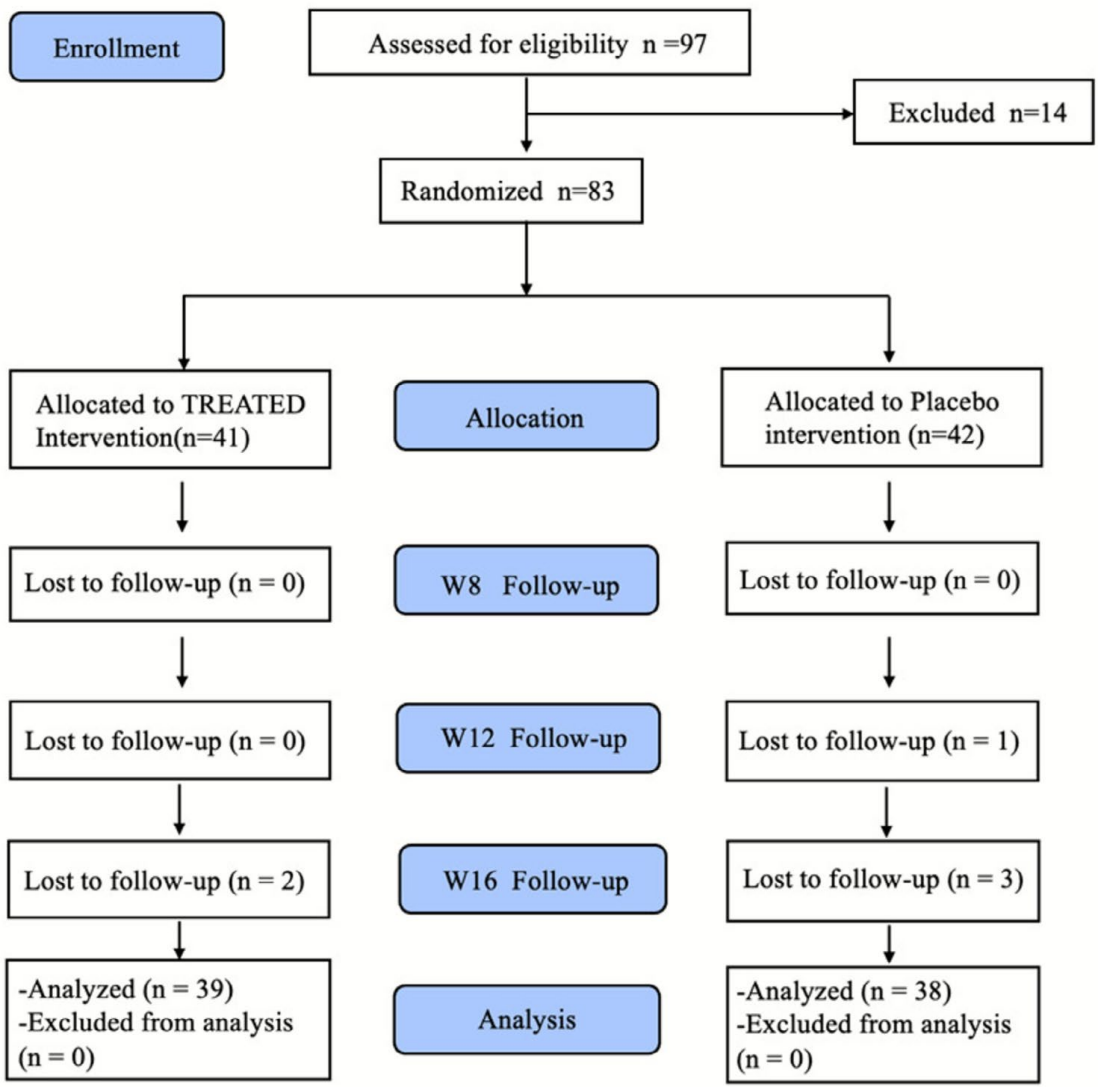
Trial recruitment and follow‐up process.

The experimental process is shown in Figure 1. A total of 97 participants were assessed for eligibility, among whom 14 were excluded. The remaining 83 participants were randomized into two groups: 41 were allocated to the treated intervention group and 42 to the placebo group. Subsequent follow‐ups were conducted at Weeks 8, 12, and 16. By the end of the study, 2 participants from the treated group and 4 from the placebo group were lost to follow‐up, respectively. In total, 39 participants in the treated group and 38 in the placebo group were included in the final analysis.

All values in Table 2 represented baseline measurements obtained prior to the collagen peptide intervention. The baseline characteristics between the placebo and treatment groups were generally well balanced. There was no significant difference in age between groups (43.85 ± 7.06 vs. 45.42 ± 6.85 years, *p* = 0.81). Although the mean BMI was significantly higher in the treatment group (23.23 ± 3.05 vs. 21.82 ± 2.09 kg/m^2^, *p* = 0.02), this difference is unlikely to confound the intervention outcomes, as variations in BMI within this range have minimal impact on skin physiology or the response to collagen peptide supplementation.

**TABLE 2:** Baseline situation of the population.

	Placebo (*n* = 38)	Treated (*n* = 39)	*p* value
Age (years)	43.85 ± 7.06	45.42 ± 6.85	0.81
Sex (Female, *n*)	38	39	/
Height (cm)	159.19 ± 4.90	158.81 ± 4.80	0.70
Weight (kg)	55.45 ± 7.20	59.48 ± 10.73	0.06
Body mass index (BMI)	21.82 ± 2.09	23.23 ± 3.05	0.02
Skin type (dry, n)	*n* = 15	*n* = 15	/
Skin type (oil, n)	*n* = 19	*n* = 20	/
Skin type (neutral, n)	*n* = 4	*n* = 4	/

### 3.2 Characterization of Bioactive Collagen Peptide Protein

The BCP sample exhibited the following molecular‐weight distribution: as a cumulative distribution, 43.01% of peptide species were ≤ 1000 Da, 59.78% were ≤ 2000 Da, and 95.09% were < 10 000 Da (Figure S1 and Table 3). The weight‐average molecular weight was approximately 2353 Da with a hydroxyproline content of 16.6% (w/w). The chromatographic profile obtained by liquid chromatography was provided in the Figure S1.

**TABLE 3:** Corresponding peak area ratios of collagen peptides with different molecular weights.

Molecular weight range (Da)	Peak area ratio %
> 10 000	4.91
5001–10 000	11.15
3001–5000	9.61
2001–3000	14.56
1001–2000	16.77
501–1000	26.08
< 500	16.93

### 3.3 The Effect of Bioactive Collagen Peptides on Facial Moisture Retention

No adverse reactions were reported by any participants throughout the trial.

**FIGURE 2:**
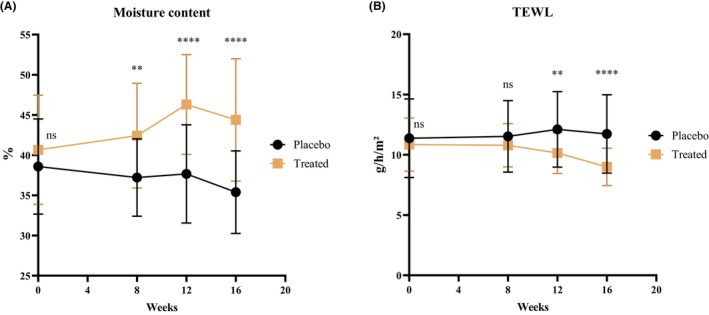
Effect of specific collagen peptides on facial moisture: (A) TEWL; (B) moisture content.

Regarding moisture content (Figure 2A)the treated group showed significantly higher moisture content than the placebo group at Week 8 post‐intervention (*p* < 0.001), and although there was a slight decrease at Week 16, the difference remained significant (*p* < 0.0001). Specifically, the baseline moisture content in the treated group was 40.66% ± 6.79%, which increased to 44.38% ± 7.62% by Week 16, reflecting an average increase of 9.15%. Meanwhile, the placebo group's moisture content decreased from a baseline of 38.58% ± 5.92% to 35.40% ± 5.14% by Week 16, showing an average decline of 8.24%.

As shown in Figure 2B, the baseline TEWL in the placebo group was 11.37 ± 3.26 g/m^2^/h, and at the end of the trial, the average TEWL increased to 11.72 ± 3.25 g/m^2^/h, representing a 3.07% increase. In contrast, the TEWL in the treated group showed an overall decreasing trend. The baseline TEWL in the treated group was 10.85 ± 2.20 g/m^2^/h, and by Week 16, it had reduced to 9.00 ± 1.55 g/m^2^/h (−17.05%). The TEWL in the treated group was significantly lower than that in the placebo group starting from Week 12 (*p* < 0.001) and was markedly lower by Week 16 (*p* < 0.0001).

These results indicated that, compared to the placebo group, the bioactive collagen peptide group showed a 20.12% decrease in TEWL and a 17.39% increase in moisture content by Week 16. BCP contributed to improved facial skin moisture retention, potentially by promoting the repair and hydration of skin structure, thereby enhancing the skin's moisturizing function.

### 3.4 Effect of Specific Collagen Peptides on Facial Texture Characteristics

**FIGURE 3:**
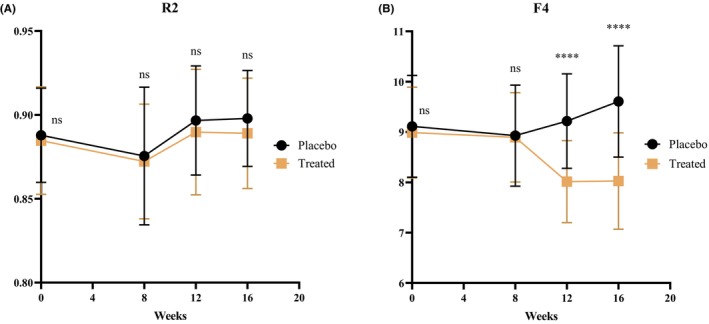
Effect of specific collagen peptides on facial elasticity and firmness: (A) facial elasticity; (B) facial firmness.

F4 reflects skin firmness, with lower values indicating greater firmness [27]. As shown in Figure 3A, no significant difference in facial elasticity (R2) was observed between the treated group and the placebo group. Regarding firmness (F4), as shown in Figure 3B, the baseline average value in the placebo group was 9.11 ± 1.01, and by Week 16, the average increased by 5.48%, suggesting reduced facial skin firmness. In contrast, the treated group had a baseline average value of 8.99 ± 0.90, which decreased to 8.06 ± 0.96 by Week 16, showing a 10.34% reduction, indicating an improvement in facial firmness. The reduction in F4 was 15.48% greater than that in the placebo group, with significant improvements observed at both Week 12 and Week 16 (*p* < 0.0001). These results suggest that continuous intake of BCP for 12 weeks can improve facial skin firmness, and this effect is sustained even after a 4‐week discontinuation period.

### 3.5 Effect of Bioactive Collagen Peptides on the Epidermis and Dermis

**FIGURE 4:**
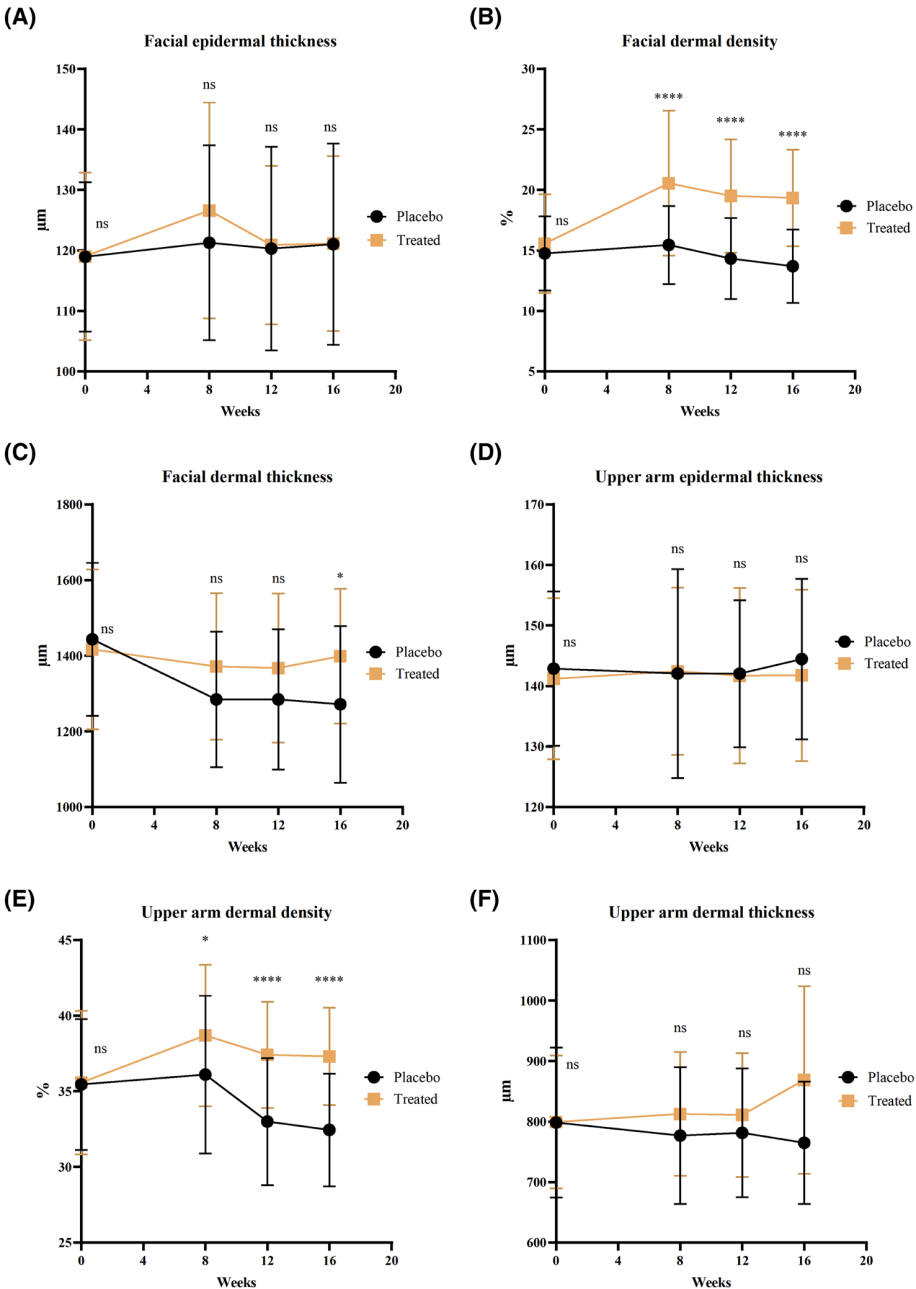
Effect of specific collagen peptides on epidermal and dermal thickness and density: (A) facial epidermal thickness; (B) facial dermal density; (C) facial dermal thickness; (D) upper arm epidermal thickness; (E) upper arm dermal density; (F) upper arm dermal thickness.

High‐frequency ultrasound technology is used to measure the epidermal and dermal thickness of the face and upper arm, while a skin imaging device analyzes skin density to investigate the effects of BCP on deep skin tissue [28]. The data indicate that after 16 weeks of intervention, no significant differences in the epidermal thickness of the face and upper arm were observed between the treated and placebo groups (Figure 4A,D), suggesting that specific collagen peptides have a limited effect on the epidermal layer. However, BCP significantly improved the dermal thickness and density of the skin.

For facial dermal thickness (Figure 4B,C), the baseline average dermal thickness in the placebo group was 1443.11 ± 202.13 μm, and by Week 16, it had reduced to 1271.45 ± 207.21 μm, representing an 11.92% decrease. In contrast, the baseline dermal thickness in the treated group was 1416.58 ± 211.51 μm, and after the intervention, it was 1398.61 ± 178.22 μm, showing a 1.27% decrease. From Week 8 onward, the dermal thickness in the treated group remained higher than that in the placebo group and was significantly greater at Week 16 (*p* < 0.05), indicating that BCP could reduce the loss of dermal thickness. Furthermore, improvements in dermal density were even more pronounced, with the treated group showing significant increases compared to the placebo group from Week 8 (*p* < 0.0001). The baseline and Week 16 average dermal densities in the treated group were 15.55% ± 4.07% and 19.34% ± 4.00%, respectively, reflecting a 19.20% increase. In the placebo group, the baseline and Week 16 dermal density values were 14.73% ± 3.07% and 13.68% ± 3.03%, respectively, showing a 7.13% decrease.

For the dermal thickness in the upper arm (Figure 4E,F), the baseline and Week 16 average thickness in the placebo group were 798.34 ± 124.03 μm and 764.87 ± 101.33 μm, respectively, reflecting a 4.19% decrease. In contrast, the baseline and Week 16 average thickness in the treated group were 799.13 ± 109.88 μm and 868.71 ± 154.95 μm, representing an 8.70% increase. Although the treated group had a higher dermal thickness in the upper arm at the beginning of the trial, no significant differences were observed. Regarding dermal density, the baseline values for the placebo and treated groups were 35.45% ± 4.33% and 35.56% ± 4.75%, respectively. By Week 16, the placebo group showed an 8.46% decrease to 32.45% ± 3.72%, whereas the treated group exhibited a 4.89% increase to 37.30% ± 3.22%. Furthermore, the dermal density of the treated group in the upper arm was significantly higher than that of the placebo group at Week 8 (*p* < 0.05), and it remained significantly higher at both Week 12 and Week 16 (*p* < 0.0001).

**FIGURE 5:**
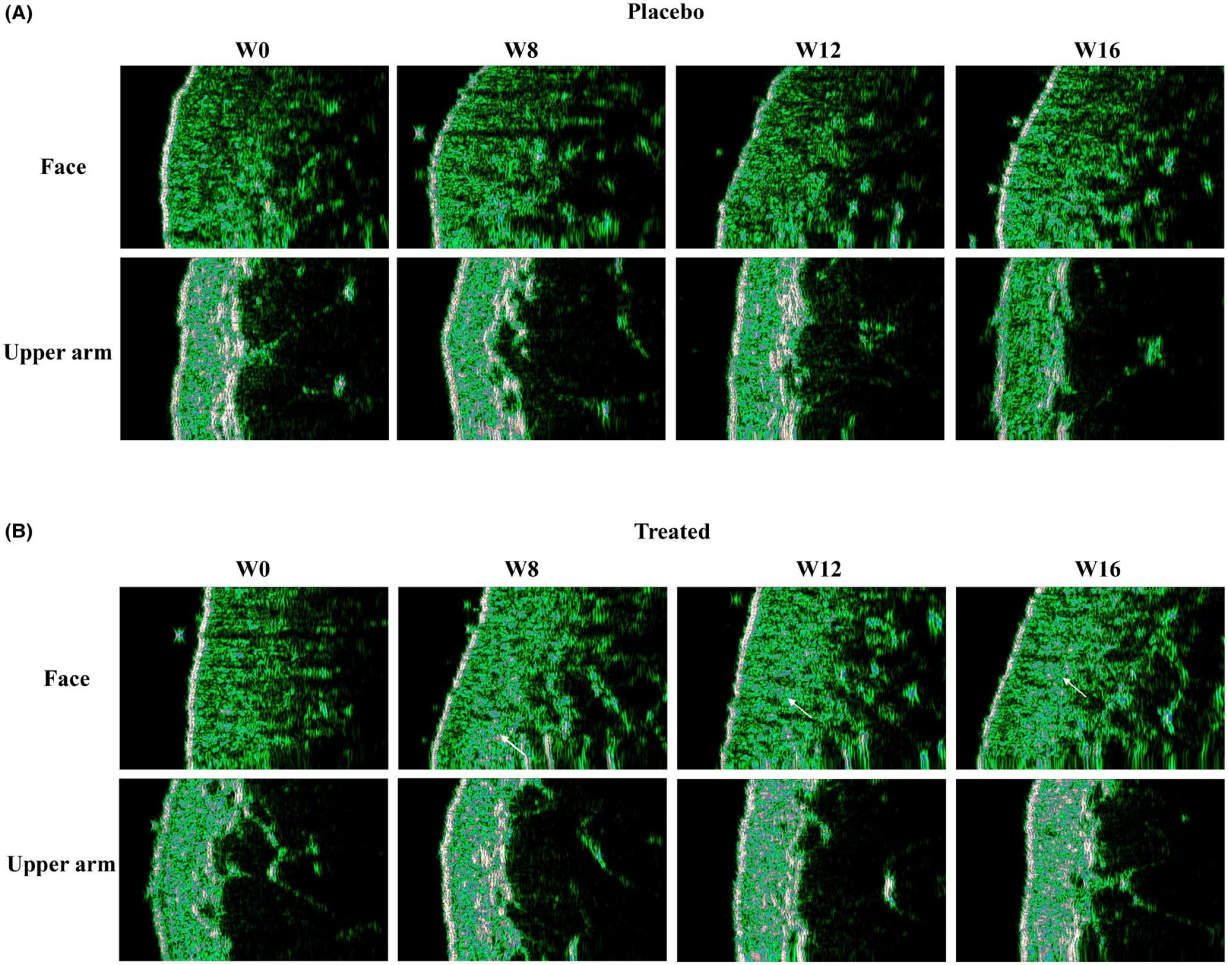
Ultrasound images of the dermis of the face and upper arms: (A) placebo group; (B) treated group.

HFUS images in Figure 5 illustrate the changes in dermal density before and after collagen peptide supplementation. An increase in dermal echogenicity was observed following the intervention, reflecting enhanced dermal structural integrity and density (as indicated by the white arrow). These qualitative findings are consistent with the quantitative measurements shown in Figure 4, further substantiating the beneficial effects of collagen peptide intake on dermal properties.

After 16 weeks of BCP supplementation, the treated group demonstrated a 10.65% increase in facial dermal thickness and a 26.33% enhancement in dermal density compared to the placebo group. BCP contributes to improved impact on the structure and function of the dermal layer, and the improvement reached statistical significance by Week 8 (*p* < 0.05). Moreover, the beneficial effects were sustained even during the 4‐week discontinuation period from Week 12 to Week 16. The dermis plays a crucial role in supporting, nourishing, and connecting the epidermis and subcutaneous tissue [29]. This result supports the possibility that the intervention improves deep skin tissue quality by enhancing dermal thickness and density.

## 4 Discussion

In this study, our BCP demonstrated a Hyp content of 16.6%, with over 40% of peptides having molecular weights < 1 kDa. As a collagen‐specific amino acid, Hyp facilitates fibroblast proliferation and dermal regeneration through HAS2 transcriptional activation [30]. Particularly, the proline‐hydroxyproline dipeptide form acts as a fibroblast growth initiator in wound healing processes [31, 32]. The 12–16 week washout period was designed based on Hyp‐containing oligopeptides' remarkable resistance to blood proteases, with previous in vivo studies showing low‐molecular‐weight collagen hydrolysates maintaining elevated skin levels for 14 days post‐administration [33]. The absorption characteristics of BCP were reflected in its molecular weight distribution, where smaller peptides (< 1 kDa) exhibited superior absorption efficiency and prolonged blood retention, with only di‐and tripeptides being absorbed via the PEPT‐1 transporter [34].

We selected vitamin C and E based on their anti‐inflammatory, antioxidant, and collagen‐stabilizing properties [35]. Vitamin C served not only as a coenzyme for prolyl 5‐hydroxylase to accelerate proline‐to‐Hyp conversion [36], but also promoted collagen triple helix formation through regulation of amino acid interactions. Synergistic effects between collagen peptides and vitamin C derivatives have been demonstrated in mitigating age‐related skin atrophy via superoxide dismutase 1 modulation [37]. Tocotrienol‐rich fractions enhance total collagen synthesis by regulating *COL I* and *COL III* gene expression [38].

Based on BCP's high hydroxyproline content and low–molecular‐weight profile (> 40% < 1 kDa), we evaluated its effects on facial skin moisture content and TEWL. Compared with placebo, subjects receiving BCP exhibited a statistically significant increase in moisture content and a reduction in TEWL (*p* < 0.05). These differences persisted at the 16‐week time point, indicating a sustained effect. We attributed these outcomes to the action of Pro‐Hyp dipeptides, which had previously been shown to stimulate fibroblast proliferation and upregulate hyaluronic acid synthesis, thereby enhancing endogenous skin moisture retention 30. Notably, this benefit appeared dose‐dependent: a prior study administering only 1650 mg/day of collagen peptides failed to improve TEWL in the forearm region [39], whereas our formulation—with its elevated Hyp content and optimized peptide size distribution—achieved significant, long‐lasting improvements in facial hydration and firmness.

Next, we evaluated skin mechanical properties. R2, representing total elasticity (Ua/Uf), did not differ significantly between the BCP and placebo groups, possibly because overall elasticity depends on dynamic and viscoelastic components and because participants' baseline R2 values were already high (> 0.8). In contrast, F4, reflecting structural resistance to negative pressure and thus skin firmness [40], was significantly increased in the BCP group compared with placebo (*p* < 0.05), indicating rapid remodeling of the dermal collagen network in the treatment cohort. Consequently, we proceeded to assess dermal structure in these participants using high‐frequency ultrasound.

HFUS at 20 MHz provides superior resolution and sufficient penetration to distinguish the dermis from subcutaneous tissue [41]. Unlike most previous trials, which seldom evaluated both dermal thickness and density [42], our study demonstrated that facial dermal density in the BCP group increased significantly by Week 8 compared with placebo, whereas a significant increase in dermal thickness was only observed at Week 16. This temporal dissociation likely reflects an early activation of fibroblast‐mediated collagen turnover—with improved fiber cross‐linking and realignment first manifesting as increased density—whereas measurable matrix deposition and thickening require a longer interval or additional cofactors. Asserin et al. similarly reported improvements in collagen network architecture after 8 weeks of oral collagen peptide intervention [28].

Notably, in a prior clinical trial using 2.5 g/day of collagen peptides, skin hydration, elasticity, and density declined by 45.8%, 38.5%, and 31.3%, respectively, during the 12–16‐week washout period 19. In contrast, neither dermal density nor thickness declined in our study, and dermal thickness even increased significantly at Week 16. These more durable and robust effects may be attributable to our formulation's higher dose, elevated hydroxyproline content, and optimized low‐molecular‐weight peptide distribution.

In the epidermal compartment, oral BCP produced no change in thickness. Lint et al., who reported that in healthy individuals' epidermal thickness is primarily age‐dependent [43], whereas topical application of a vitamin C–squalene conjugate significantly increased epidermal thickness via upregulation of type III collagen peptides [44]. These data suggest that while oral collagen peptides effectively remodel the dermal matrix, targeted topical formulations may be required to modulate epidermal architecture.

Study limitations include a modest sample size (*n* = 77) from specific demographics, potentially limiting generalizability given environmental and genetic influences on collagen supplementation outcomes. Safety in this study was assessed through dermatological and physical evaluations without blood routine or biochemical monitoring, and efficacy was evaluated using non‐invasive functional and imaging techniques rather than molecular assays. These approaches inevitably limit the mechanistic depth of our findings. Future studies incorporating laboratory monitoring and molecular endpoints are warranted to provide more comprehensive evidence.

While meta‐analyses indicate collagen peptides' cardiovascular benefits (significant fat mass reduction, *p* = 0.010; LDL reduction, *p* = 0.048) [45]. In the context of sports health, collagen peptides have been frequently cited for their beneficial effects on joint and bone integrity [46, 47, 48, 49, 50], their ability to enhance adult musculoskeletal performance [51], and their role in reducing muscle soreness [52]. Clinical outcomes appear dose‐ and composition‐dependent. Future studies should investigate Hyp content, molecular distribution, and dosage effects.

This randomized, controlled, double‐blind, placebo trial demonstrates that 12‐week oral BCP supplementation followed by a 4‐week washout significantly improves skin biophysical properties including thickness, density, elasticity, and hydration. The findings establish correlations between BCP's Hyp content/molecular profile and clinical efficacy, providing a scientific foundation for optimizing collagen peptide formulations and clinical evaluation protocols.

## Author Contributions

Conceptualization and methodology, Yue Zhou, Weixing Zhu and Wenyu Luo; investigation and formal analysis, Weixing Zhu, Wenyu Luo; writing – original draft, Yu Wang; writing – review and editing, Yu Wang, Yanfeng Ma, Wenyu Luo; supervision, Weixing Zhu, Wenyu Luo.

## Ethics Statement

This study involving human participants was reviewed and approved by the Ethics Committee of Shanghai Yingke Testing Technology Co. Ltd. (Approval No.: YKEC‐2024‐001). The study adhered to Good Clinical Practice guidelines and the Declaration of Helsinki.

## Consent

Informed consent was obtained from all participants involved in the study.

## Conflicts of Interest

Yu Wang, Yanfeng Ma, and Yue Zhou are employees of Shanghai Meifute Biotechnology Co. Ltd. Weixing Zhu and Wenyu Luo declare no conflicts of interest. The study was conducted collaboratively between academic and clinical institutions, and all analyses and interpretations were performed independently and objectively.

## Supporting information


**Figure S1:** Collagen peptide gel chromatography liquid phase peak diagram.

## Data Availability

Data available on request due to privacy/ethical restrictions.
