# Supplementary material for: The Sustained Effects of Bioactive Collagen Peptides on Skin Health: A Randomized, Double‐Blind, Placebo‐Controlled Clinical Study
Source: J Cosmet Dermatol. 2025 Nov 28;24(12):e70565. doi: 10.1111/jocd.70565 (PMC12661388; doi:10.1111/jocd.70565)
Supplement: Supplementary file 1 — Figure S1: Collagen peptide gel chromatography liquid phase peak diagram. [file JOCD-24-e70565-s001.pdf]

Supplementary Material 1

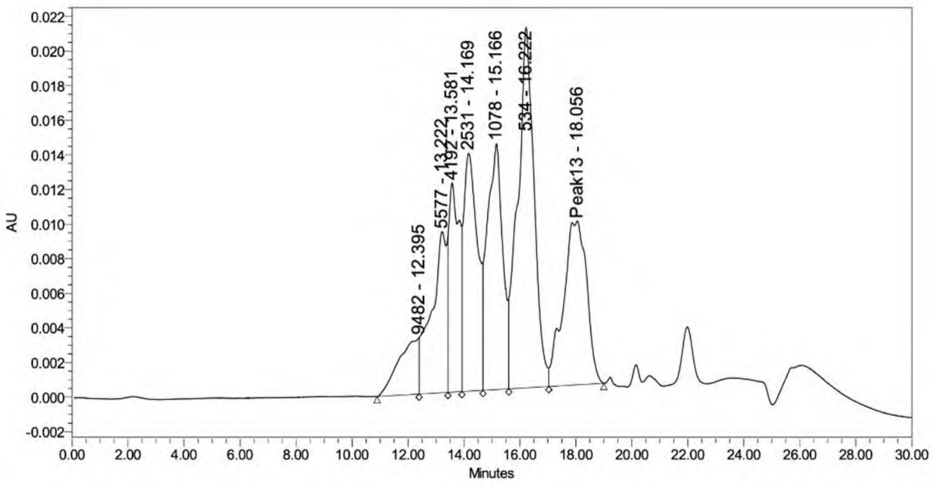

Supplement Figure1. Collagen peptide gel chromatography liquid phase peak diagram
